# Selection of suitable candidate genes for miRNA expression normalization in Yellow River Carp (*Cyprinus carpio*. var)

**DOI:** 10.1038/s41598-019-44982-x

**Published:** 2019-06-18

**Authors:** Fang Wang, Qian-wen Yang, Wen-Jie Zhao, Qi-Yan Du, Zhong-Jie Chang

**Affiliations:** College of Life Science, Henan Normal University, Xinxiang, Henan 453007 People’s Republic of China

**Keywords:** miRNAs, Marine chemistry

## Abstract

Yellow River carp is widely cultivated in the world due to its economic value in aquaculture, and the faster growth of females compared to males. It is believed that microRNAs (miRNA) are involved in gonadal differentiation and development. qPCR is the most preferred method for miRNA functional analysis. Reliable reference genes for normalization in qRT-PCR are the key to ensuring the accuracy of this method. The aim of present research was to evaluate as well as identify the efficacy of reference genes for miRNA expression using qRT-PCR in Yellow River carp. Nine ncRNAs (*miR-101*, *miR-23a*, *let7a*, *miR-26a*, *miR-146a*, *miR-451*, *U6*, *5S*, and 18*S*) were chosen and tested in four sample sets: (1) different tissues in adult carp, (2) different tissues in juvenile carp, (3) different early developmental stages of carp, and (4) different developmental stages of carp gonads. The stability and suitability values were calculated using NormFinder, geNorm, and BestKeeper software. The results showed that *5S* was a suitable reference gene in different tissues of adult and juvenile carp. The genes *5S*, 18*S*, and *U6* were the most stable reference genes in the early developmental stages of carp. *Let-7a* and *miR-23a* were considered as the suitable reference genes in the development of gonads. All these reference genes were subsequently validated using *miR-430*. The results showed that genes *5S* and 18*S* were the most suitable reference genes to normalize miRNA expression under normal growth conditions in early different developmental stages. The genes *Let-7a*, and *miR-23a* were the most suitable in different developmental stages. The present study is the first comprehensive study of the stability of miRNA reference genes in Yellow River carp, providing valuable as well as basic data for investigating more accurate miRNA expression during gonadal differentiation and development of carp.

## Introduction

*Cyprinus carpio*, common carp, is one of the most important cyprinid species, which accounts for 10% of freshwater global aquaculture production^1^. As a member of them, as species, Yellow River carp has stable traits, fast growth, and high economic value.

MicroRNAs (miRNAs) are a class of non-coding small RNA molecules, which is 20–24nt, play important regulatory roles in many organisms (e.g., viruses, plants, worms, fish and mammals)^2–4^, and in various biological processes (e.g., growth, embryonic development, immune, apoptosis, cell proliferation, and differentiation, cell cycle control and stress responses of various organs)^5–9^. MiRNAs bind to target mRNAs through base paring with 3′ un-translated regions leading to mRNA cleavage, or to translational repression, which depends on the degree of complementarity of base pairs between miRNA and its target^10,11^. Because miRNAs have such important functions, they are being widely studied in various species and in different fields.

Accurate miRNA expression levels are important to elucidate their roles. Several experimental methods can be used for profiling miRNAs (e.g., northern blot^12^, microarrays^13^, RAKE assays^14^, real-time PCR, or quantitative RT-PCR^15^). Quantitative RT-PCR (qRT-PCR) is an accurate and sensitive technique for analyzing miRNA levels^16^. qRT-PCR is widely used by people detecting miRNA expression in many organisms^17–19^. Normalization of miRNA levels in this method depends on the stability of the expression of reference genes^20^. Wong *et al*. demonstrated that it is the best way to normalize target RNA levels with reference genes belonging to the same RNA class^21^. Thus, the small non-coding RNA family (ncRNA; for example small nuclear RNA, snRNA; and small nucleolar RNA) is recommended for miRNA.

Several candidate reference RNA genes have been tested under different experimental conditions, and in different tissues, to identify suitable endogenous reference genes^22,23^. The most widely-used reference genes for miRNA quantification are small nuclear RNAs, for example *U5 snRNA* and *U6 snRNA*^24^, and ribosomal RNAs like 18*S rRNA*, 5*S rRNA*, and 5.8*S rRNA*^25^. Protein-coding genes have also been evaluated as reference genes for miRNA expression analysis by qRT-PCR^26,27^. However, studies have shown that there are no genes that do not vary after changing the experimental conditions in different tissues. Thus, it is necessary to deeply evaluate the expression of candidate genes for every experimental condition and tissue, before performing experiments.

Only a few studies have analyzed the stability of some miRNAs in human, porcine and rat tissues^28–30^. Several studies have analyzed suitable reference genes for measuring the level of miRNA expression by qRT-PCR in plant^26,31,32^. In teleost fish, few reports have explored the stability of miRNAs to be used as reference genes. Xu *et al*. investigated the stability of seven miRNAs in different tissues and developmental stages of grass carp through qRT-PCR^33^, but there are no reports on this field in common carp.

To identify suitable reference genes in Yellow River carp in a comprehensive way, expression stability of nine candidate reference genes (which had been investigated as reference genes in partial reports) was explored in early developmental stages (in which the gonads have not yet differentiated), and in different tissues of juvenile carp and adult carp. We also analyzed the expression of nine candidate reference genes in gonads of juvenile and adult carp (in which the gonads had already differentiated), and also in primordial gonads, which is the beginning of gonadal development and a critical period of sex determination and differentiation.

## Results

### qRT-PCR amplification validations

All primers were tested for specificity by standard curve analysis, using serial dilutions (100%, 50%, 20%, 10%, 5%, 2%, 1%) of cDNA as templates in Real-Time RT-PCR. Amplification efficiency was 92–105% (Table 1); melting curves showed a single peak. These results demonstrated that the PCR reactions are high quality and specificity. Thus, these reference genes were used for the analysis. In addition, each gene produced a single band of the expected size in 1% agarose gel electrophoresis.

**Table 1:** Sequence information for nine selected candidate reference genes.

gene	Sequence (5′ → 3′	Amplification efficiency
*miR-101*	TACAGTACTGTGATAACTGAG	0.997
*mi-23a*	CATCACATTGCCAGGGATTTC	0.995
*miR-26a*	CGTTCAGTATCCAGGATAGGCT	0.999
*miR-146a*	CGTGAGACTGATTCCATAGATG	0.998
*let-7a*	CGGTGAGGTAGTAGGTTGTATAGTT	0.990
*miR-4*5*1*	AAACCGTTACCATTACTGAGTT	0.992
18*S*	GGACACGGAAAGGATTGACAG	0.998
	CGGAGTCTCGTTCGTTATCGGC	
5*S*	GCGTAGAGGAAGCACACCAAT	0.999
	TTCCGCAGGAGGTCCCCTACAG	
*u6*	ACAGAGAAGATTAGCATGGCC	0.990
	GACCAATTCTCGATTTGTGCG	

### Expression levels and stability of reference genes in different adult carp tissues

**Figure 1:**
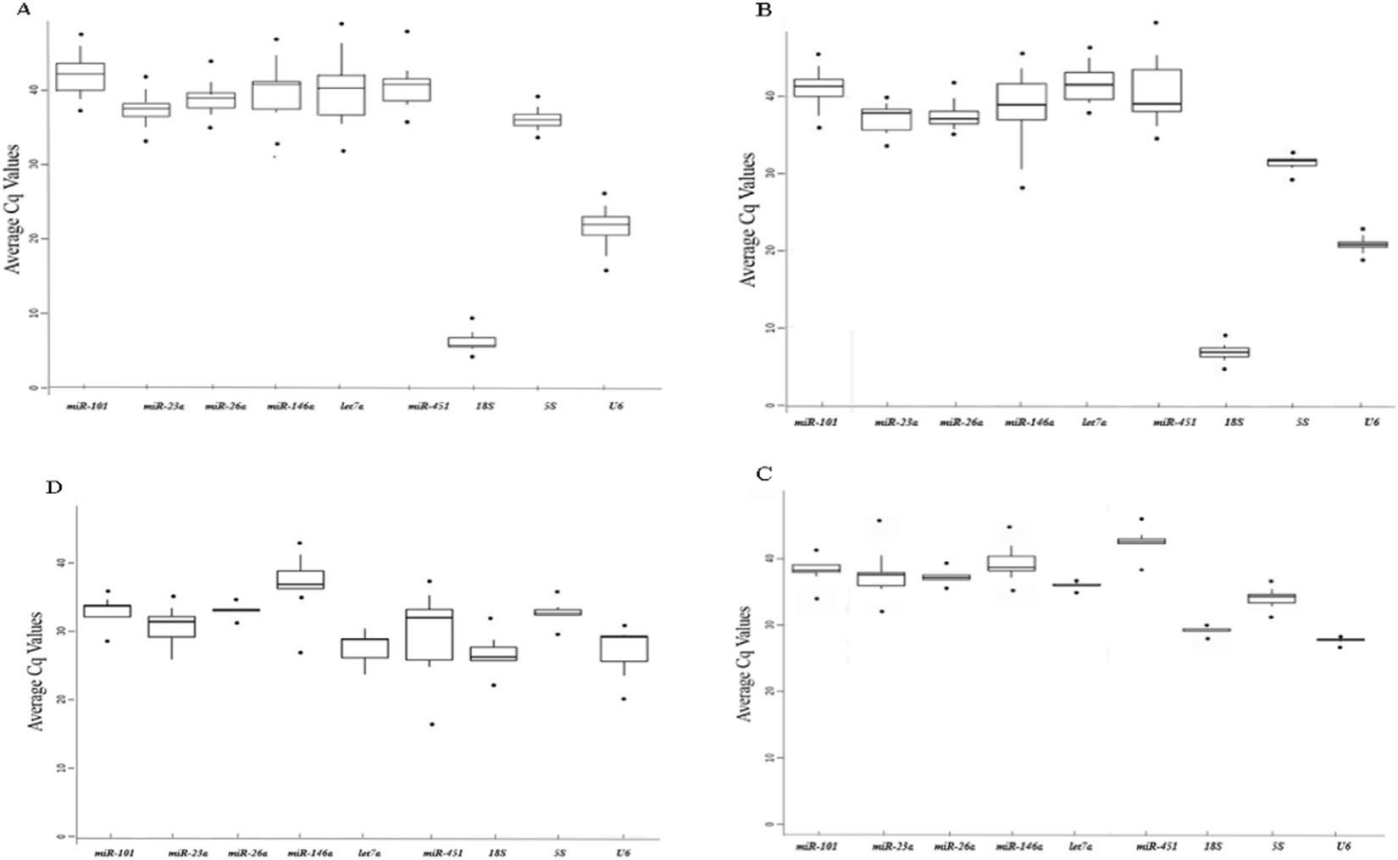
Expression levels of the nine potential reference genes in Yellow River carps. (**A**) Different tissues of adult, (**B**) different tissues of juvenile, (**C**) early development stages, (**D**) development of gonads. Data is shown as average Cq values ± SD (n = 6).

Minor changes in general expression levels and mean Cq values of nine reference genes were found in the RNA level in various tissues of adult carp. As shown in Fig. 1A the Cq values for the nine reference genes ranged from 5.40 to 45.92. *MiR-101a* was the reference gene with the lowest expression (Cq values ranged from 40 to 45.92). The gene 18*S* was the most highly expressed reference gene with the lowest Cq value (from 5.40 to 11.13). *U6* is a small nuclear RNA that is frequently used to normalize miRNA data; it also showed a high expression (Cq values ranged from 18.05 to 24.52).

To investigate the suitability of the nine reference genes for miRNA expression normalization, we calculated their stability values by geNorm, NormFinder, and BestKeeper. The gene *5S* showed the highest stability in the three software programs, and in the different tissues of adult carp. The gene 18 *s* was the most unstable according to NormFinder and BestKeeper. while it was ranked third with geNorm. *U6* was the most stable gene according to geNorm. while ranked eighth and fifth with NormFinder and BestKeeper respectively.

**Figure 2:**
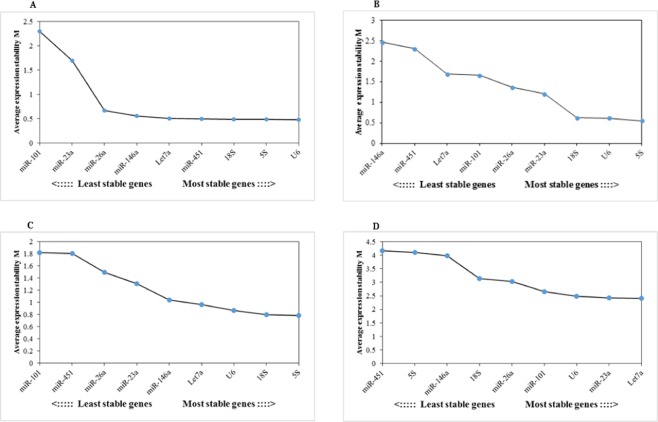
Average expression stability values (*M*) calculated by geNorm. (**A**) Different tissues of adult, (**B**) different tissues of juvenile, (**C**) early development stages, (**D**) development of gonads.

The most stable reference gene has the lowest M value, while the least stable one has the highest M value. Our result showed that all the reference genes had low M values (<1.5) except *miR-101* and *miR-23a*. According to NormFinder and BestKeeper, *5S* was the best candidate gene, followed by *miR-23a* (Table 2 and Fig. 2A). Therefore, *5S* was recommended as the best candidate gene for analysis of X in adult Yellow River carps.

**Table 2:** Rank order of each candidate reference gene in different tissues of adult.

Ranking order (better-good-average)									
method	1	2	3	4	5	6	7	8	9
Bestkeeper	*5* *S*	*miR-23a*	*miR-26a*	*miR-101*	*miR-451*	*Let7a*	*miR-146a*	*U6*	18 *S*
Genorm	*U6*	5 *S*	18 *S*	*miR-4*5*1*	*Let7a*	*miR-146a*	*miR-26a*	*miR-23a*	*miR-101*
NormFinder	*5* *S*	*miR-23a*	*miR-26a*	*miR-101*	*U6*	*Let7a*	*miR-451*	*miR-146a*	18 *S*

### Expression levels and stability of reference genes in different tissues of juvenile carp

The Cq values of the nine reference genes ranged from 5.85 to 45.31, across the different tissues examined in juvenile carp. *MiR-451* was the gene with the lowest expression (Cq values ranging from 36.14 to 45.31), followed by *Let-7a* and *miR-101*. The gene 18 *s* was the most highly expressed reference gene with the lowest Cq value (5.85–11.32). *U6* had Cq values ranging from 18.78 to 22.82 (Fig. 1B).

The stability of the nine reference genes was calculated by geNorm, NormFinder, and BestKeeper. The gene *5S* was the most stable; 18 *s* and *miR-146a* were the least stable in different juvenile carp tissues. GeNorm showed that all the reference genes had low M values (<1.5), except for *miR-101*, *Let-7a, miR-451*, and *miR-146a*. The gene *5S* was the most stable, according to geNorm, followed by *U6* and 18 *s*. The gene 18 *s* was third for stability according to geNorm, but it was the least stable according to NormFinder and BestKeeper. *MiR-23a* was second in stability according to BestKeeper, was fourth in stability in geNorm, and third in stability in NormFinder. *MiR-26a* was the second in stability in NormFinder, fifth in stability in BestKeeper and geNorm, and NormFinder (Table 3 and Fig. 2B).

**Table 3:** Rank order of each candidate reference gene in different tissues of juvenile.

Ranking order (better-good-average)									
method	1	2	3	4	5	6	7	8	9
Bestkeeper	5 *S*	*miR-23a*	*miR-101*	*U6*	*miR-26a*	*Let7a*	*miR-451*	*miR-146a*	18 *S*
Genorm	5 *S*	*U6*	18 *S*	*miR-23a*	*miR-26a*	*miR-101*	*Let7a*	*miR-451*	*miR-146a*
NormFinder	*5* *S*	*miR-26a*	*miR-23a*	*miR-101*	*U6*	*Let7a*	*miR-451*	*miR-146a*	18 *S*

### Expression levels and stability of reference genes in early different developmental stages

**Figure 3:**
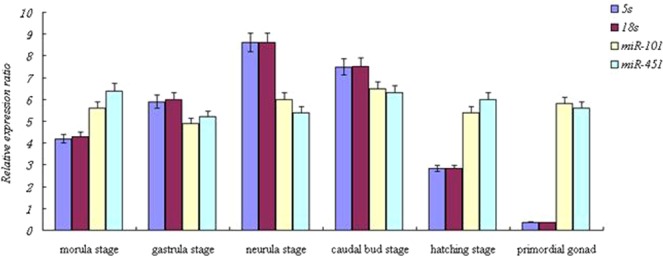
Expression profile of *miR-430* and validation of selected reference genes in early different development stages of Yellow River Carp. Error bars represent the mean of three technical replicates ± SD.

To investigate the suitability of the nine reference genes for miRNA expression normalization in different early developmental stages, samples of morula, gastrula, neurula, caudal bud, hatching, and primordial gonad were analyzed. As shown in Fig. 3C, the Cq values for the nine reference genes ranged from 47.26 to 27.7. *U6* was the most highly expressed reference gene with the lowest Cq values (27.7 to 28.26). The gene 18 *s* had Cq values ranging from 29.2 to 29.96. *MiR-451* was the gene with the lowest expression (with Cq values ranging from 38.32 to 45.97), followed by *miR-146a*, and *miR-101*.

Normfinder, BestKeeper, and geNorm demonstrated that *5S* and 18 *s* had the highest stability, while *miR-101* was the most unstable. The *5S* and 18 *s* were the best candidate genes in NormFinder and geNorm, followed by *U6* and *Let-7a*. GeNorm showed that all the reference genes had low M values (<1.5), except for *miR-451* and *miR-101*. According to BestKeeper, *U6* was the best gene for X, followed by 18 *s* and *Let-7a*. The gene *5S* was first in NormFinder and geNorm, and fourth in BestKeeper. Therefore, *5S*, 18 *s*, and *U6* were considered the best reference genes in different developmental stages of Yellow River carp (Table 4 and Fig. 2C).

**Table 4:** Rank order of each candidate reference gene in different tissues of early development stages.

Ranking order (better-good-average)									
method	1	2	3	4	5	6	7	8	9
Bestkeeper	*U6*	*18* *S*	*Let7a*	5 *S*	*miR-4*5*1*	*miR-146a*	*miR-23a*	*miR-26a*	*miR-101*
Genorm	*5* *S*	18 *S*	*U6*	*Let7a*	*miR-146a*	*miR-23a*	*miR-26a*	*miR-4*5*1*	*miR-101*
NormFinder	*5* *S*	18 *S*	*U6*	*Let7a*	*miR-146a*	*miR-23a*	*miR-26a*	*miR-4*5*1*	*miR-101*

### Expression levels and stability of reference genes during development of carp gonads

**Figure 4:**
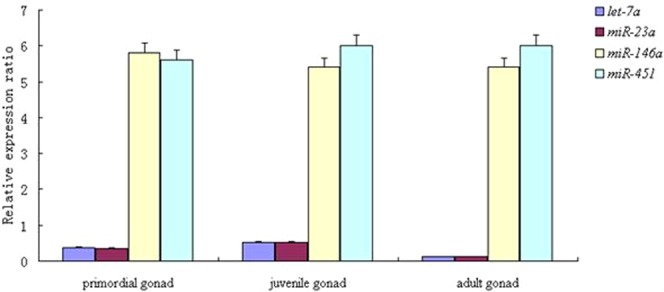
Expression profile of *miR-430* and validation of selected reference genes during development of Yellow River Carp gonads. Error bars represent the mean of three technical replicates ± SD.

To analyze the expression of the nine reference genes during gonadal development of carp, expression was measured in the gonad at three developmental periods: primordial gonad (undifferentiated period), juvenile carp, and adult carp. As shown in Fig. 4D, the Cq values ranged from 22.12 to 38.81. The gene *U6* showed relatively high abundance (Cq values from 23.51 to 29.44), followed by 18 *s* (Cq values from 22.12 to 28.76). The expression level of *U6* and 18 *s* was lower in the gonad than in other tissues.

*Let-7a* was the most stably expressed gene according to geNorm, followed by *miR-23a* and *U6*. In geNorm all the reference genes had high M values (>1.5), which was different from the results of other samples. Normfinder showed that *miR-23a* was the most stable gene, followed by *Let-7a*. The gene *5S* was the most stable reference gene in BestKeeper, followed by *miR-10*, but it was the second least stable according to geNorm, and the least stable according to NormFinder. *MiR-451* was the least stable gene during the development of carp gonads, according to both BestKeeper and geNorm. *Let-7a* and *miR-23a* were thus considered the best reference genes during gonadal development of Yellow River carp (Table 5 and Fig. 2D).

**Table 5:** Rank order of each candidate reference gene in development of gonads.

Ranking order (better-good-average)									
method	1	2	3	4	5	6	7	8	9
Bestkeeper	*5* *S*	*miR-101*	18 *S*	*miR-23a*	*Let7a*	*U6*	*miR-26a*	*miR-146a*	*miR-451*
Genorm	*Let7a*	*miR-23a*	*U6*	*miR-101*	*miR-26a*	18 *S*	*miR-146a*	5 *S*	*miR-451*
NormFinder	*miR-23a*	*Let7a*	*miR-101*	*U6*	18 *S*	*miR-26a*	*miR-146a*	*MiR-4*5*1*	*5* *S*

### Validation of selected reference genes by *miR-430* expression analysis

To validate the X of potential reference genes in common carp, the relative expression of *miR-430* (a miRNA potentially involved in sex in Yellow River carp) was normalized using the most stable reference gene, the multiple best reference genes, and the least stable reference gene, in different early developmental stages, and in different gonad developmental stages. Our previous research showed that *miR-430* was significantly down-regulated during early developmental stages. Data normalizations using the most stably expressed reference genes, *5S* and 18 *s*, as well as stable reference genes in combination, resulted in consistent *miR-430* expression patterns during the early developmental stages. By contrast, the expression of *miR-430* was considerably biased when the least stably expressed reference gene *miR-101* was used for data normalization (Fig. 3). Similar results were observed when *miR-430* was compared to the stable reference genes *Let-7a* and *miR-23a* during gonad development, and stable reference genes in combination. The results normalized using the most unstable reference gene *miR-451* showed no significant differences between primordial gonad and juvenile carp (Fig. 4). The above results indicated that the most stable reference genes and most suitable combination showed similar levels of miRNA expression, so that they could be used for normalization of miRNA in Yellow River carp. The least stable reference genes failed to standardize the expression data effectively.

## Materials and Methods

### Sample collection

Research was preformed preformed according to the Animal Experimental Guidelines of the Ethical Committee of The University of China. The Yellow River carp (*Cyprinus carpio* var.) used in this study was obtained from Henan academy of fishery science, Zhengzhou (Henan, P. R. China) and maintained at the Genetic Laboratory (Henan Normal University, Xinxiang, P. R. China) in through-flow water tanks at 25 ± 2 °C under natural photoperiod for an initial acclimation period. Fish were fed twice daily during the preparation of experiment, and no fish died during the experiment. Embryos were obtained by natural spawning and cultured in embryo medium following standard procedures. Juvenile carp (one year old) and adults (two years old) were selected randomly.

Eleven tissues including forebrain, hindbrain, muscle, foregut, hindgut, kidney, liver, spleen, heart, gill and fin were taken from three juvenile carps and from three adults in aseptic conditions. The embryos and early larvae were sampled at six developmental stages (12 specimens per samples from each stage): morula, gastrula, neurula, caudal bud, hatching, and primordial gonad (an important period in which the expression of many miRNAs in the gonads changed significantly according to our previous study; data not shown). Gonads were collected from juvenile and adult carp(three males and three females). Each stage had three biological replicates. All samples were immediately frozen in liquid nitrogen and stored at −80 °C for further use.

The Yellow River carp used in this study were obtained from the aquaculture base of Henan Normal University and cultured in our own laboratory. All fish were anesthetized with 2-phenxyethanol before being euthanized. Fish were cared for according to the Regulations for the Administration of Affairs Concerning Experimental Animals for the Science and Technology Bureau of China. The experimental protocols were approved by the Animal Management Committee of Henan Provincial Laboratory.

### Selection of candidate reference genes and design of primers

Nine candidate reference genes including *miR-101*, *miR-23a*, *miR-26a*, *miR-146a*, *let7a*, *miR-451*, *U6*, *5S* and 18*S* were selected as reference genes for miRNA expression normalization by qRT-PCR, due to their wide use in the literature.

Primers for the nine selected candidate reference genes are listed in Table 1. Primers were designed using Primer Premier 5.0, according to the rules reported in previous studies^34^. Primers for miRNAs were designed according to the principle of stem-sloop primers^35,36^. Forward primers were designed according to miRNA sequences (sequences from high-throughput sequencing results). The reverse primer was a universal primer (5′-GTGCAGGGTCCGAGGT-3′).

### RNA isolation and cDNA synthesis

Total RNA was extracted using TRIzol reagent following the manufacturer’s instructions (Invitrogen, Carlsbad, USA); it was then treated with RNase-free DNasel (Promega, Wisconsin, USA) to remove contaminating DNA. The RNA concentration and purity was determined using a Nanodrop 2000 spectrophotometer (Nanodrop Technologies, Wilmington, DE) measuring absorbance at 260 nm and 280 nm. We also checked the degradation level of RNA samples by gel electrophoresis. RNA samples with ratio at OD260/280 over 1.95 and no degradation were used for further experiment. Reverse transcription was performed using the one-step PrimeScript miRNA cDNA Synthesis Kit (TakaRa). Reverse primers for RT-PCR and stem-loop primers were used for miRNAs and for non-miRNA ncRNAs, respectively.

### QRT-PCR

qRT-PCR was performed in 96-well blocks with a PikoReal Real-time PCR System (Thermo Fisher Scientific) using the SYBR Premix Ex TaqTM Kit (TaKaRa Biotechnology). Reactions were performed with 2 of diluted cDNA, 200 nM of each primer (Invitrogen) and 10 μL of 2 × SYBR PCR Master Mix with the following amplification conditions: 95 °C for 10 min, and 45 cycles of 95 °C for 15 s and 60 °C for 1 min. All measurements were performed using three biological replicates. Negative RT control, negative poly (A) polymerase controls, and non-template controls were also included in each run.

### Stability expression and data analysis

The stability of candidate reference genes was assessed with three programs: geNorm^36^, NormFinder^37^, BestKeeper^38^. GeNorm (v.3.5) calculates a gene expression stability value (*M*) for a control gene, as the average pairwise variation V for that gene with all other reference genes^36^. NormFinder (v.20) is an application for determining the optimal normalization gene(s) among various candidates. It uses a variance (ANOVA) estimation approach that ranks the reference genes based on intra- and inter-group variations, and conforms to their expression stability^39^. BestKeeper (v.1.0) is a program that calculates standard deviation, percentage of covariance, and coefficient of correlation from the Cq values, to determine the expression stability of reference genes.

### Reference gene validation

To confirm the reliability of the potential reference genes, *miR-430*, a putative sex-involved miRNA, was selected from previous research, because it exhibited high expression during gonad differentiation and development in Yellow River Carp^40^. The expression of *miR-430*, was normalized with both the most stable genes, and multiple of the most and least stable gene in different early developmental stages and in different gonad developmental stages. The primers are listed in Table 1. Three biological replicates and three technical replicates were used for each sample. The conditions of qRT-PCR amplification were same as those described above for X. The relative expression of all genes was computed based on the 2-ΔΔCt method as described by Livak *et al*.^41^.

In addition, the qPCR efficiency results were stated using the software LinRegPCR.

### Ethics approval

The Yellow River carp used in this study were obtained from the aquaculture base of Henan Normal University and cultured in our own laboratory, and all fish were anesthetized with 2-phenxyethanol before being euhanized. The fish were cared for according to the Regulations for the Administration of Affairs Concerning Experimental Animals for the Science and Technology Bureau of China throughout the study, and the experimental protocols were approved by the Animal Management Committee of Henan Provincial Laboratory.

## Discussion

miRNAs were increasingly reported as regulators in organisms that might play very important roles in many biological processes^42,43^. QRT-PCR is the most common method for analyzing the expression profiles of miRNAs. However, accuracy and reliability of qRT-PCR results depend on the use of suitable reference genes as internal controls to normalize miRNA expression levels^20,44^.

In teleost, few studies have been conducted to validate reference genes. Johnston *et al*. were the first to conduct miRNA expression analysis in a teleost fish^45^. Xu *et al*. investigated the stability of seven miRNAs in different tissues and different developmental stages of grass carp^3^3. Diego *et al*. conducted a comprehensive evaluation of the characteristics of reference genes in gonad samples of turbot (*Scophthalmus maximus*) at different development times reared at different temperatures^46^.

As we known, the determination of suitable reference genes in common carp (Yellow River carp) has not been reported previously.

In this study we used three common statistical approaches (NormFinder, BestKeeper and geNorm) to assess the stability of expression of nine candidate reference genes, in order to identify the best reference genes for different tissues, different developmental stages, and different gonads in Yellow River carp. In Xu’s study^33^, *miR-101a* and *miR-451* were also selected to investigate; *MiR-101a* was the most stable miRNA. Our results indicate that *5S* is the best reference gene for different tissues of adult and juvenile common carp. Xu’s results showed that the tissue type is an important variability factor for miRNA expression stability, which is in agreement with our study.

When early developmental stages were considered, the most suitable genes were *5S*, *18* *s*, and *U6*, according to the three statistical approaches; the least stable genes were *miR-101* and *miR-451*. In embryonic developmental stages of Chinese Perch (*Siniperca chuatsi*), *miR-101a* was the most stable reference miRNA^47^, but was the least stable gene in the present study. The gene *18* *s* was the least stable in all tissues, while it was the second stale gene in early developmental stages. In addition, *miR-101*, and *miR-451* were the least stable genes in different tissues in early developmental stages. In Xu’s study *miR-126-3p* was the most stable reference miRNA gene during developmental stages 1–5, while *miR-22a* was the most stable during developmental stages 6–18^33^. Together, these findings are consistent with the fact that the developing carp is changing across developmental time. Pinhal *et al*. showed that the complexity of miRNA expression in Nile tilapia increases during development and differentiation^48^; they compared later developmental stages and adult stages. A limited number of miRNAs was expressed during early developmental stages; they suggest that increasing complexity of miRNA expression profiles may trigger development and differentiation of specific cells and tissues. The results of NormFinder, BestKeeper, and geNorm are almost identical when early developmental stages were considered.

Differences in gender may lead to differences in gene expression stability. In Pinhal’s study, the expression of miRNA was also influenced by sex^48^. Our results showed that when the gonad was considered as a whole, the results are different from those in different tissues and developmental stages. Our results showed that *miR-23a* and *Let-7a* the most stable genes by NormFinder and geNorm. *5S* being ranked third analyzed for stability by BestKeeper, while ranked ninth by NormFinder and eighth by geNorm. These observations are compatible with the fact that the stability of reference genes can change when factors like sex are considered.

*5S* and *U6*, which act as housekeeping ncRNAs, are frequently used in many miRNA studies to normalize miRNA data. In teleost studies, *U6* and *18* *s* are the most common genes used for normalization^49–51^. The gene *5S* was selected as the reference control for normalizing miRNA expression in marine medaka and Paralichthys olivaceus^23,52,53^. In our study, *5S* was the best reference gene in different tissues and in early developmental stages of common carp. There were only slight differences between the results obtained by the three algorithms. During gonad development BestKeeper identified *5S* as the most stable. These results suggest that *5S* is a suitable reference gene for different tissues in early developmental stages of common carp. *U6* has been used as endogenous control in human solid tissues^23^, and is stable in somatic embryogenic and adult tissues^32^. *U6* has been considered one of the least stable genes in the liver^54^. In the present study, *U6* ranked first or third in the early development of common carp with slight differences between the results obtained by the three algorithms. While ranked second and middle in different tissues and third, fourth, and seventh in the gonad with significant differences among the results determined by the three algorithms, which indicates that *U6* may be a suitable reference gene for common carp in early developmental stages, but may not be suitable for different tissues and gonads. Xu *et al*. discarded *18* *s* from their study on grass carp because it exhibited altered expression^33^. The gene *18* *s* was second according to the three algorithms in early developmental stages, however it was among the least stable reference genes in different tissue samples and gonad samples.

The genes *18* *s* and *U6* were highly expressed in all the samples; they had the highest expression in different tissues, but relatively low expression in the gonad and in early developmental stages. This may be related to their function during gonadal development of common carp.

The geNorm program is used to assess reference genes by calculating the gene expression stability value M. The most stable reference gene has the lowest M value, while the least stable gene has the highest M value, and the M value should also be lower than 1.5^42^. In this study, geNorm stability values in gonad samples were greater than 1.5, which indicates that sex is an important variability factor that affects the stability of miRNA expression.

Our results demonstrate that the three reference gene determination methods show conflicting outcomes, especially in samples of developing gonads. For example, *5S* was the most stable reference gene in BestKeeper, while it was the second least stable by geNorm, and the least stable gene by NormFinder analysis. Diego *et al*. offered a comprehensive evaluation of the characteristics of reference gene selection methods. They found that NormFinder had the best approaches for reference gene selection, while geNorm results proved to be unreliable on the other side^46^. So, according to our analysis, *5S* was the most stable gene in all experimental conditions, except in gonad samples.

## Conclusion

This study tested the suitability of nine ncRNA genes in multi-tissue experiment, early developmental stages, and gonad of Yellow River carp with three commonly-used programs. The gene *5S* is the best option for X in multi-tissue experiments. The genes *5S* and 18 *s* are suitable reference genes in early developmental stages, and *Let-7a* and *miR-23a* are suitable reference genes during gonad development. These results highlight the importance of identifying suitable reference genes for normalization of miRNA expression under different experiment conditions. Our study analyzed the expression stability of several candidate genes deeply and comprehensively to determine the suitability reference genes in Yellow River carp. These results will be helpful for the accurate determination of miRNA expression in future common carp studies.
